# Different rates of (non-)synonymous mutations in astrovirus genes; correlation with gene function

**DOI:** 10.1186/1743-422X-4-25

**Published:** 2007-03-07

**Authors:** Formijn J van Hemert, Vladimir V Lukashov, Ben Berkhout

**Affiliations:** 1 Laboratory of Experimental Virology, Department of Medical Microbiology, Center for Infection and Immunity Amsterdam (CINIMA), Academic Medical Center, University of Amsterdam, The Netherlands

## Abstract

**Background:**

Complete genome sequences of the *Astroviridae *include human, non-human mammalian and avian species. A consensus topology of astroviruses has been derived from nucleotide substitutions in the full-length genomes and from non-synonymous nucleotide substitutions in each of the three ORFs. Analyses of synonymous substitutions displayed a loss of tree structure, suggesting either saturation of the substitution model or a deviant pattern of synonymous substitutions in certain virus species.

**Results:**

We analyzed the complete *Astroviridae *family for the inference of adaptive molecular evolution at sites and in branches. High rates of synonymous mutations are observed among the non-human virus species. Deviant patterns of synonymous substitutions are found in the capsid structural genes. Purifying selection is a dominant force among all astrovirus genes and only few codon sites showed values for the dN/dS ratio that may indicate site-specific molecular adaptation during virus evolution. One of these sites is the glycine residue of a RGD motif in ORF2 of human astrovirus serotype 1. RGD or similar integrin recognition motifs are present in nearly all astrovirus species.

**Conclusion:**

Phylogenetic analysis directed by maximum likelihood approximation allows the inclusion of significantly more evolutionary history and thereby, improves the estimation of dN and dS. Sites with enhanced values for dN/dS are prominent at domains in charge of environmental communication (f.i. VP27 and domain 4 in ORF1a) more than at domains dedicated to intrinsic virus functions (f.i. VP34 and ORF1b (the virus polymerase)). Integrin recognition may play a key role in astrovirus to target cell attachment.

## Background

Human astrovirus has been recognized as the second most common cause of diarrhoea among children under 5 years old [1]. In animal and bird farms, an astrovirus infection is fatal for a considerable part of the livestock [2,3]. The family of *Astroviridae *is divided in two genera: *Mamastrovirus *(mammalian astroviruses) and *Avastrovirus *(avian astroviruses). The pathogen is a non-enveloped virion with a single-stranded, positive-sense RNA of approximately 6.8 kb in size [4]. The virus genome contains three open reading frames designated ORF1a (2.8 kb), ORF1b (1.6 kb) and ORF2 (2.4 kb). Translation of ORF1b depends on translation of ORF1a by a ribosomal frame-shift mechanism [5]. The primary translation products are processed into the virus protease (ORF1a) and the virus polymerase (ORF1b). ORF2 encodes a structural protein that is intracellularly processed at the C-terminal part by caspase protease, by which genome packaging and virus particle release is promoted [6]. As part of the released virus particle, ORF2 protein is processed further by trypsin to acquire the mature capsid proteins VP34 and VP27/25, a process accompanied by a considerable increase of virus infectivity [7]. Consensus on the post-translational processing routes of ORF1a, ORF1b and ORF2 polyproteins has not yet been attained [8,9].

An inventory of evolutionary relationships among astroviruses has been published confirming the topology that is generally accepted for the astroviruses and pointing to a strong selection against non-synonymous substitutions [10]. Phylogenetic analyses based solely on the synonymous substitutions resulted in loss of tree structure due to the shortening of specific ancestral branches in trees of all ORFs. Avian, sheep, and human virus species appeared to be virtually equidistant in these trees. Such a loss of tree structure or tree compression may be interpreted either as the result of saturation of synonymous substitutions or to a peculiar pattern of synonymous substitutions that is typical for specific members of the astrovirus family. To address this issue, we analyze the astrovirus genes by means of nucleotide substitution models based on maximum likelihood approximation because these models are better suited for the estimation of mutational rates in highly divergent genes than models relying on the Jukes-Cantor correction for multiple hits at the same site.

Phylogenetic analysis by maximum likelihood (PAML3.14) [11] offers a set of sophisticated models to assess the extent of (non-) synonymous substitutions in genes. The current status of the PAML programs displays a profound documentation for the inference of sites and branches prone to molecular adaptation and is supported by validated statistics [12-14]. For instance, adaptive evolution is observed in the hemagglutinin gene of human influenza virus type A [15].

Applying PAML to the genes of the complete astrovirus family, we show that tree compression (shortening of ancestral branches) can be ascribed to deviant rates of synonymous mutation at discrete regions of ORF2 in certain astrovirus species. Tree compression is absent in ORF1a and ORF1b as revealed by PAML due to its ability to include more evolutionary history. Sites that tend to escape from purifying selection correlate to protein domains dedicated to environmental communication rather than to replication and assembly of the virus. Finally, we propose that integrin recognition of ORF2 domains plays a key role in the process of cell binding by astrovirus.

## Results

### (Non-) synonymous substitutions in astrovirus genes: branch models

**Figure 1:**
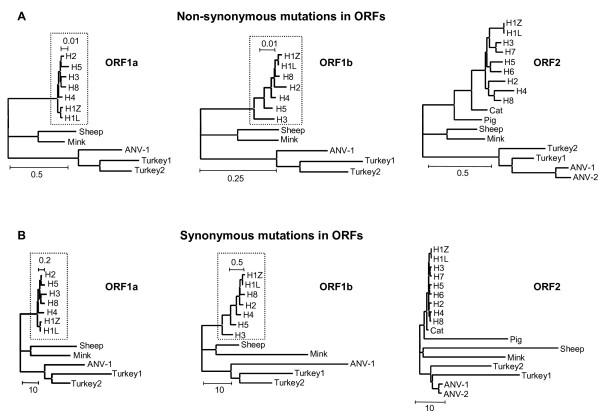
**PAML trees of non-synonymous (A) and synonymous (B) substitutions in ORF1a (left panel), ORF1b (middle panel) and ORF2 (right panel) of astrovirus**. PAML trees have been constructed by decorating the topology supplied by the input treefile with the branch lengths estimated by means of PAML approximation. The scale bars indicate the relative extent of mutational rates as (non-) synonymous substitutions per (non-) synonymous site corrected for multiple hits at the same site.

The values for dN/dS of the branch model applied to the astrovirus ORFs are all far below the value of 1 (Table 1), by convention considered as the lower limit for positive selection. In fact, dN/dS values do not exceed 0.16, except for ORF2 in the cat and human4 isolates (*0.1904 *and *0.2610*, respectively). Obviously, purifying selection is very dominant in branches specifying astrovirus species. The individual values of dN and dS may be more easily interpreted in a tree format (Fig 1) than as raw data in a table. Unrooted dN and dS trees are part of PAML's output and have been constructed by pasting the dN and dS values from Table 1 as branch length onto the amino acid tree topology supplied to PAML as input tree file.

**Table 1:** Values for (non-)synonymous mutational rates and dN/dS ratios in astrovirus genes

**Species**		**ORF1a**			**ORF1b**			**ORF2**		
TipBranche	AccNum	dN	dS	dN/dS	dN	dS	dN/dS	dN	dS	dN/dS

Human1	Z25771	0.0040	0.0445	0.0899	0.0026	0.0775	0.0335	0.0049	0.0503	0.0974
Human1	L23513	0.0031	0.0228	0.1360	0.0000	0.0130	0.0000	0.0057	0.0386	0.1477
Human2	L13745	0.0047	0.0514	0.0914	0.0086	0.1326	0.0649	0.1334	0.8681	0.1537
Human3	AF141381	0.0085	0.2177	0.0390	0.0143	0.3686	0.0388	0.0415	0.5710	0.0727
Human4	DQ070852	0.0068	0.2338	0.0291	0.0033	0.1517	0.0218	0.1564	0.5992	*0.2610*
Human5	DQ028633	0.0122	0.1390	0.0878	0.0061	0.1872	0.0326	0.0959	0.7401	0.1296
Human6	Z46658							0.0876	0.7478	0.1171
Human7	Y08632							0.0582	0.5862	0.0993
Human8	AF260508	0.0075	0.1508	0.0497	0.0033	0.1802	0.0183	0.0536	0.4412	0.1215
Sheep	Y15937	0.3386	28.9094	0.0117	0.1369	8.2335	0.0166	0.2367	46.6085	0.0051
Mink	AY179509	0.2348	24.9745	0.0094	0.1357	32.7666	0.0041	0.2994	28.4143	0.0105
Cat	AF056197							0.1262	0.6628	*0.1904*
Pig	AB037272							0.2204	28.9632	0.0076
Turkey1	Y15936	0.3483	37.4061	0.0093	0.2029	18.8746	0.0107	0.1717	30.1582	0.0057
Turkey2	AF206663	0.2821	11.8241	0.0239	0.1604	11.5272	0.0139	0.3652	20.9202	0.0175
ANV	AB033998	0.3863	25.2068	0.0153	0.1692	41.9092	0.0040	0.1007	1.3416	0.0751
ANV2	AB046864	0.1208	1.0567	0.1143						

The virus isolates available for analysis of ORF1a, ORF1b and ORF2 are mentioned in the first column and specified by their accession numbers. With respect to ORF2, one representative per human serotype was included. Separate values for dN and dS of the species' tip branches as well as their ratios are presented for each ORF, individually. Values for dN/dS exceeding 0.15 are in *italics*. Ancestral branches were not included.

The dN trees of the three ORFs (Fig 1A) are in close agreement with the widely accepted astrovirus phylogeny [10,16-21]. The ORFs evolve independently and mutational rates are lower in ORF1b than in ORF1a or ORF2 as indicated by the different scale bars. Also, the relative lengths of the individual branches mimic those in trees inferred by other means. For instance, the sequence of ORF2 in human astrovirus serotype 4 is known as the most distant among the human ORF2 sequences [19]. Apparently, the alignment attained by the multi-step MUSCLE procedure (see Materials & Methods) displayed an average accuracy and is at least as good as more laborious ClustalW based protocols, despite the sequence diversity typical for the astrovirus data set.

Synonymous rates of mutation in astrovirus genes differ from non-synonymous mutational rates by as much as one to two orders of magnitude as indicated by the dN/dS ratio in Table 1 and the scale bars in Fig 1B. Nevertheless, dS trees of ORF1a and ORF1b based on synonymous substitutions are close to the corresponding trees derived from non-synonymous substitutions or amino acid replacements. Loss of tree structure [10] or tree compression caused by the virtual disappearance of ancestral branches can hardly be observed illustrating the power of the substitution model to incorporate multiple hits at the same site. In contrast, loss of tree structure and shortening of ancestral branches is observed in the dS tree of ORF2 and appears to be due to enhanced accumulation of synonymous substitutions in ORF2 sequences of the astroviruses of pig, sheep, mink and turkey compared to viruses in humans, cats and both avian nephritis viruses.

**Figure 2:**
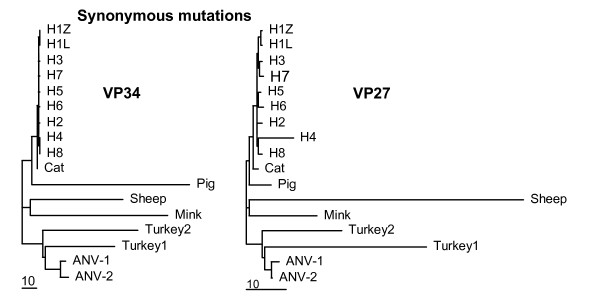
**PAML trees of synonymous substitutions in the astrovirus capsid proteins VP34 (left panel) and VP27 (right panel)**. See text and legend to Fig 1.

The ORF2 regions coding for the virus capsid proteins VP34 and VP27 may differ in the accumulation of synonymous substitutions. To test this, the ORF2 alignment was divided in two portions based on the cleavage by trypsin. VP34 is slightly more conserved than VP27, but most ORF2 values are close to the average of the corresponding values for VP34 and VP27 (Table 2). Indeed, dN trees of VP34 and VP27 (not shown) are nicely deep-rooted and nearly identical to the dN tree of the complete ORF2 (Fig 1A). By contrast, dS values of sheep virus capsids increase from 36.5 in VP34 to 64.4 in VP27, while these values decrease from 53.3 to 12.3 in mink. In pig, the difference is even more dramatic, 60.6 in VP34 and only 4.8 in VP27. Both turkey isolates exchange their dS values of about 20 and 33 for VP34 and VP27, respectively. Trees with values for synonymous substitutions pasted as branch lengths onto the same topology more clearly show the species-specific differences in synonymous mutational rates between VP34 and VP27 (Fig 2). With respect to VP34, tree compression is due to high values of dS in pig, sheep, mink and turkey, similar to the results for ORF2. In the dS tree of VP27, however, the sheep virus and to a lesser extent both turkey viruses are responsible for tree compression. The viruses of pig and mink are essentially conforming the human, feline and avian viruses. Apparently, the VP34 and VP27 domains tolerate considerable differences with respect to the extent of synonymous mutational rates as estimated by means of PAML. Similar to ORF1a and ORF1b, the domains VP34 and VP27 of ORF2 display mutually independent patterns of molecular evolution.

**Table 2:** Values for (non-)synonymous mutational rates and dN/dS ratios in astrovirus capsid protein genes

**Species**		**VP34**			**VP27**		
TipBranche	AccNum	dN	dS	dN/dS	dN	dS	dN/dS

Human1	Z25771	0.0063	0.0418	0.1507	0.0018	0.0701	0.0257
Human1	L23513	0.0030	0.0430	0.0698	0.0100	0.0219	0.4566
Human2	L13745	0.0267	0.5843	0.0457	0.2687	1.0243	0.2623
Human3	AF141381	0.0132	0.6429	0.0205	0.0670	0.5253	0.1275
Human4	DQ070852	0.0066	0.1794	0.0368	0.3372	7.8626	0.0429
Human5	DQ028633	0.0253	0.7471	0.0339	0.1589	0.6257	0.2540
Human6	Z46658	0.0276	0.5950	0.0464	0.1544	1.0149	0.1521
Human7	Y08632	0.0306	0.3748	0.0816	0.0827	0.8255	0.1002
Human8	AF260508	0.0011	0.2941	0.0037	0.1026	0.9374	0.1095
Sheep	Y15937	0.1203	35.7089	0.0034	0.3946	60.6402	0.0065
Mink	AY179509	0.1615	52.9013	0.0031	0.5333	14.9279	0.0357
Cat	AF056197	0.0223	0.4164	0.0536	0.2495	1.0615	0.2350
Pig	AB037272	0.1595	60.6407	0.0026	0.2829	4.6804	0.0604
Turkey1	Y15936	0.1673	27.0532	0.0062	0.1803	35.9325	0.0050
Turkey2	AF206663	0.2719	37.0278	0.0073	0.5306	17.7786	0.0298
ANV-1	AB033998	0.0831	1.9230	0.0432	0.1215	1.7471	0.0695
ANV-2	AB046864	0.1228	2.9662	0.0414	0.1172	0.2508	0.4673

The viral species available are mentioned in the first column and specified by their accession numbers. One representative per human serotype was included. See also text and legend to Table 1.

Currently, it cannot be excluded that even PAML encounters saturation-related problems at high rates of mutation in a properly aligned set of sequences [22]. However, this may only be a partial explanation to the large variation of synonymous mutational rates in adjacent domains of specific astrovirus genes and genomes.

### Site models: dN/dS values in relation to domain functions

**Figure 3:**
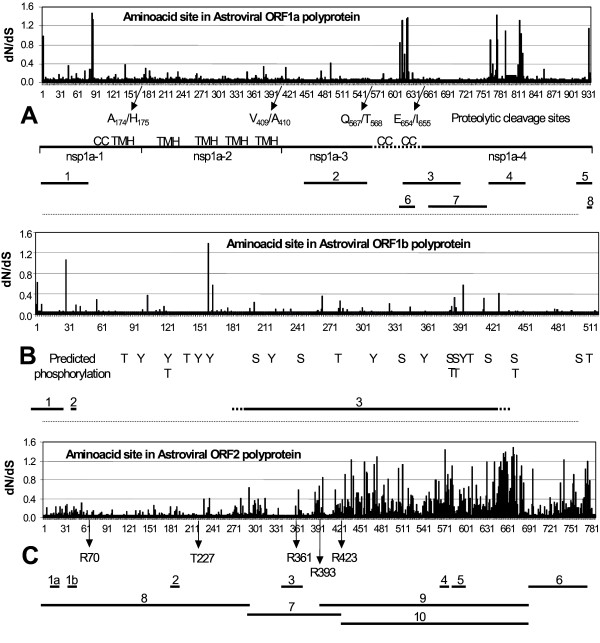
**Values for dN/dS of amino acid sites in relation to domain functions in astrovirus ORF1a (A), ORF1b (B) and ORF2 (C)**. For each site, the BEB-derived value of dN/dS (Y-axis) is plotted against the position of the residue in the polyprotein encoded by the ORF (X-axis). Arrows and scale bars indicate sites proposed for proteolytic digestion and predicted for phosphorylation (T, Y and S in ORF1b) as well as domain functions described in publications or predicted by servers. Numbering is according to human astrovirus serotype 1 Z25771. **3A**: CC: coiled coil, TMH: transmembrane helix Nsp1a: non-structural protein 1a. 1: Region putatively involved in viral RNA helicase activity (1–80). 2: Protease domain (447–589). 3: Reported death domain and nuclear localization signal (620–714). 4: Hypervariable region and cell-growth induced deletion (760–838). 5: Ribosomal frameshifting signal (918–935). 6: Variable region prone to O-glycosylation and phosphorylation (608–632). 7: Putative VPg region associated with viral RNA replication (664–757). 8: KKXX-like ER retention (931–934). **3B**: 1: Ribosomal frameshifting signal (1–28). 2: Predicted furin-type cleavage site (32–38). 3: Conserved polymerase domain (...-...). **3C: **Rxxx: Arg-residues reported to mark tryptic digestion. T227: Thr-residue reported to bind viral RNA. 1a & 1b: NGR, RGD-like cell attachment motif (16–52). 2: LDV, RGD-like cell attachment motif (182–184). 3: Reported common epitope (340–376). 4: RGD: integrin recognition motif for cell attachment (572–574). 5: Predicted serotypic epitope (580–606). 6: Region of intracellular caspase digestion (701–787). 7: Region of extracellular tryptic digestion (300–423). 8, 9, 10: VP34, VP27, VP25, viral capsid proteins.

Site models of CODEML allow for the estimation of dN/dS ratios at individual codon sites in an alignment of sequences. Because of the skewed astrovirus population – large distances between the few clades and overrepresentation of closely related human virus species – statistical support was taken only from the Bayes-Empirical-Bayes (BEB) output [14]. With respect to ORF2 of the human virus species, we confined the analysis to one virus representative per human serotype (except for H1). All three ORFs display sites that tend to escape from purifying selection (Table 3). Posterior mean values of dN/dS for BEB-selected sites are between 1 and 1.5 in all ORFs with high standard errors and low posterior probabilities. Conventionally, a dN/dS value of 1 marks the transition from neutral evolution to weakly positive selection. BEB-selected sites (dN/dS = 1) are not distributed randomly in ORF1a and ORF2 but appear to cluster. This is more easily observed when all sites are plotted as a string of local dN/dS values of the ORF's codons superimposed on a map of virus functions embedded in the ORF as described in literature and/or predicted by servers (Fig 3). In ORF1a (Fig 3A), clusters with relatively high dN/dS values are located around amino acid 620, at position 775, 777 and 812–817. The 614–624 cluster maps near the N-terminus of the nsp1a-4 domain. The exact position of this N-terminus is still under debate, being either T568 or I655 [8,23]. A coiled coil, a nuclear localization signal and a death domain for the induction of cellular apoptosis are found in this region [24]. Also, this part of ORF1a displays enhanced amino acid variability and may be prone to O-glycosylation and phosphorylation. The BEB-selected sites between 775–814 mark the borders of the interspecies hypervariable region (760–838) that also harbors the peptide that is deleted by cell culture adaptation of the virus [25]. At the C-terminus, the ribosomal frame-shift signal is decorated with a BEB-selected threonine residue next to a predicted motif for retention at the endoplasmic reticulum. As expected from the branch model analysis (see above), sites with dN/dS values less than 0.1 constitute a large majority in ORF1a.

**Table 3:** Sites prone to positive selection in astrovirus genes

**Bayes Empirical Bayes (BEB) Analysis**					
**BEB positively selected sites in ORF1a**					

Position in Alignment	Amino acid residue	Position in H1Z25771	Posterior mean value for dN/dS	± SE for dN/dS	Posterior probability (dN/dS > 1)

3	Y	3	0.988	0.576	0.522
109	K	86	1.474	0.225	0.951
110	E	87	1.336	0.407	0.817
812	A	614	1.316	0.421	0.796
813	T	615	1.322	0.416	0.801
821	S	623	1.357	0.385	0.835
822	A	624	1.383	0.351	0.855
1060	V	755	1.436	0.290	0.911
1078	S	790	1.087	0.581	0.623
1102	P	814	1.326	0.410	0.804
1105	P	817	1.036	0.585	0.575
1263	T	932	1.157	0.503	0.646

**BEB positively selected sites in ORF1b**					

Position in Alignment	Amino acid residue	Position in H1Z25771	Posterior mean value for dN/dS	± SE for dN/dS	Posterior probability (dN/dS > 1)

45	L	29	1.068	0.802	0.603
178	N	160	1.384	0.860	0.704

**BEB positively selected sites in ORF2 (selection)**					

Position in Alignment	Amino acid residue	Position in H1Z25771	Posterior mean value for dN/dS	± SE for dN/dS	Posterior probability (dN/dS > 1)

489	A	440	1.231	0.418	0.660
507	D	458	1.182	0.439	0.611
533	V	472	1.123	0.457	0.551
537	L	476	1.282	0.396	0.717
604	S	513	1.122	0.499	0.582
676	G	573	1.441	0.264	0.901
686	T	583	1.094	0.478	0.537
716	K	612	1.078	0.468	0.507
745	S	633	1.182	0.430	0.603
755	S	643	1.183	0.430	0.606
764	H	652	1.172	0.438	0.599
768	S	656	1.350	0.357	0.797
774	N	658	1.269	0.411	0.710
791	T	659	1.389	0.329	0.845
793	Y	661	1.182	0.424	0.603
800	A	668	1.292	0.391	0.729
802	Q	670	1.481	0.205	0.953
803	F	671	1.333	0.366	0.775
804	D	674	1.316	0.394	0.767
814	I	681	1.090	0.470	0.528
882	I	741	1.219	0.431	0.653
919	N	775	1.187	0.442	0.621

The Bayes-Emperical-Bayes (BEB) output of the CODEML NSsites model 8 for nucleotide substitution allows the selection of codon sites (as amino acids) with a posterior mean value for dN/dS ≥ 1 and provides a standard error as well asa value for posterior probability. The position of the selected amino acid in the alignment has been converted into the location in the H1Z25771 polyprotein. "Selection" in the ORF2 panel refers to the limitation of one representative per human serotype in ORF2 analyses.

The dN/dS distribution in ORF1b illustrates the power of purifying selection even more dramatically (Fig 3B). Nearly all sites in the conserved polymerase domain have dN/dS values far below 0.25 to 0.3 being the transition region from purifying selection to neutral evolution. Outside the conserved domain, but within the virus polymerase, the asparagine residue at position 160 shows a posterior mean value of 1.384 for dN/dS, but with weak statistical support. The same holds for the leucine residue 29 (dN/dS = 1.068), between the frameshift stem-loop and the predicted furin-type cleavage site. All sites possibly prone to phosphorylation in ORF1b are subjected to strong purifying selection.

The dN/dS profile of ORF2 polyprotein (Fig 3C) clearly shows the distinction between capsid functions either dedicated to virus replication and assembly or involved in environmental communication. The N-terminal VP34 protein is involved in the packaging of virus RNA. Replacement of the much conserved threonine residue at position 227, for instance, abolishes the formation of virus particles due to loss of the ability to bind virus RNA [26]. Nearly all sites in mature VP34 are under strong purifying selection, indicating the involvement of VP34 in conserved virus functions. Mature VP25/27 encoded by the C-terminal part of ORF2 constitutes the virus' spike protein and carries the region of serotypic antibody recognition (580–606). Variation is beneficial for immune escape and sequence homology decreases to levels that locally even hamper a proper alignment of the available data set. As a result, dN/dS values tend to increase towards neutral evolution. The limited data set prevents a statistical discrimination between sites with dN/dS values = 1 due to either weak but bona fide positive selection or merely site-specific heterogeneity. However, clusters of sites with dN/dS values exceeding 1 can be observed. The region 440–476 contains four amino acid residues with dN/dS values of about 1.2 and constitutes the N-terminal part of VP25. At the C-terminus of VP25 (633–681), 12 amino acids display dN/dS values = 1, with Q670 having the highest dN/dS value of 1.481 ± 0.205 with a posterior probability of 0.953. Finally, two residues (I741 and N775) with dN/dS values of about 1.2 reside in the C-terminal part of ORF2. Proteolysis by caspase of this region is the first step in the maturation process of the ORF2 polyprotein [6]. RNA packaging and cellular release cannot occur without cleavage by caspase, which in turn is probably activated by the death domain in ORF1a.

### Cell attachment by integrin recognition

The glycine residue at position 573 in ORF2 is among the top three of all sites having a dN/dS value of 1.441 ± 0.264 and a posterior probability of 0.901. Surprisingly, this Gly573 is identified by PROSITE as the core residue of a RGD tripeptide being a recognition sequence for cellular attachment to an integrin-type cell surface receptor [27]. It seems a paradox that a site prone to adaptive molecular evolution also participates in an important replication function. However, integrin binding can also be fulfilled by similar motifs, for instance KGD, RHD, NGR and LDV, and may be enhanced by synergy sites like the tripeptide RNS [28]. RNS, KGD and RHD oligopeptides are absent in the collection of ORFs2, but the integrin-binding motifs RGD, LDV and NGR are present in astrovirus ORF2 except for Turkey1 (Table 4). Most of these putative integrin-binding sequences are located in VP34 rather than in the spike protein VP25. The LDV tripeptide is located at position 183 and NGR is present (sometimes even in duplicate) at the positions 17 and 51 of VP34 (H1Z25771 numbering).

**Table 4:** Integrin-binding sequence motifs in astrovirus capsid proteins

**Integrin-binding sequence motifs in Astroviral capsid proteins**					
CorePeptide	IntegrinType	CorePeptide	IntegrinType	CorePeptide	IntegrinType

**RGD**	α5,8,vβ1	**LDV**	α4β1	**NGR**	RGD-like
	αvβ 3,5,6,8				low affinity
	αIIbβ3				
AstroVirus	location	AstroVirus	location	AstroVirus	location
H1	VP25	H1	VP34	H1	VP34
Mink	VP34	H3	VP34	H2	VP34
Sheep	VP34	H4	VP34	H3	VP34
		H5	VP34	H4	VP34
		H6	VP34	H5	VP34
		H7	VP34	H6	VP34
		H8	VP34	H7	VP34
		Feline	VP34	H8	VP34 & VP25
		Sheep	VP34	Feline	VP34
		Turkey2	VP25	Pig	VP34
				Turkey2	VP25

Sequence motifs (in boldface) are mentioned with the specified subtypes of integrin. The presence of the RDG consensus sequence in human astrovirus serotype 1 was revealed by means of PROSITE. Other putative sequence motifs were traced by text-editing and as yet, experimental support is absent.

## Discussion

Previous research showed that avian astrovirus species displayed different topologies in trees based on either synonymous or non-synonymous substitutions suggesting a deviant pattern of synonymous substitutions specifically in these species [10]. More specifically, tree compression due to shortening of ancestral branches caused loss of resolution among the non-human mammalian and the avian species in all ORFs leaving solely the human serotypes properly resolved. Recently, we demonstrated a switch in the recent evolution of *Astroviridae *driving the synonymous codon usage in genes of specifically the non-human mammalian viruses towards the mean codon usage in genes of their hosts [21]. The present study employs phylogenetic analysis by maximum likelihood approximation to assess the extent of (non-) synonymous mutational rates at the expense of tree-building capacity. Fortunately, there is consensus in literature on the phylogenetic topology of astrovirus species derived from amino acid replacements or non-synonymous nucleotide substitutions [10,16-21]. This provides the opportunity to decorate a tree carrying this consensus topology with the branch lengths estimated by PAML for synonymous or non-synonymous substitutions in astrovirus ORFs. By these means, we obtained standard-like trees without significant compression for astrovirus ORF1a and ORF1b, despite the large extent of synonymous substitutions in the non-human species. Apparently, substitution models subjected to maximum likelihood approximation tolerate considerably higher levels of mutational saturation than "classic" substitution models relying on the Jukes-Cantor correction for multiple hits at the same site and hence allow the inclusion of significantly more evolutionary history during phylogenetic analysis. Improvement of dN and dS estimation is the result.

With respect to astrovirus ORF2, the tree based on non-synonymous substitutions is very much standard-like, whereas the tree based on synonymous substitutions clearly suffers from compression due to the extended branch lengths of pig, sheep, mink and turkey astrovirus species. A bipartition of ORF2 into the two regions encoding the VP34 and VP27 capsid proteins shows that the branch extension of these species is observed in the VP34 domain, but confined to sheep and the turkeys in VP27. In mink and particularly pig, enhanced rates of synonymous mutation are present in the VP34 domain, but absent in the VP27 domain of ORF2. At present, we cannot offer a proper explanation for this species- and domain-specific enhancement in the rates of synonymous mutation. The consistency of dN trees with data in literature [10,16-20] argues against an improper alignment of the astrovirus sequences. It is conceivable that the substitution model applied reaches its limit at a certain level in the rate of mutation. However, VP34 carries the majority of elongated branch lengths, but is slightly better conserved than its ORF2 colleague VP27. The avian clade may pose a biological argument relevant to the problem. As shown above, turkey astroviruses consistently do and avian nephritis viruses do not display branch length extension indicating a possibly relevant difference between these species. Investigation at the source of the sequences involved has pointed out that the sequences of turkey species have been determined by RT-PCR of virus RNA extracted directly from stools and organs [18,29], whereas RNAs of avian nephritis virus have been prepared from cell-culture supernatants after three consecutive rounds of plaque purification before being subjected to RT-PCR amplification [30]. It has already been mentioned that adaptation of human astroviruses to grow in continuous cell lines induces a 45-nucleotide deletion near the 3'-end of ORF1a [25]. In coronavirus, mutations have been associated with isolation and passage in primate cell lines [31,32]. In conclusion, selection at the level of isolation and purification as well as mutation at the level of propagation may affect the difference between the rates of synonymous and non-synonymous substitution in astrovirus genes. Future research may address this issue.

The relationship between negative and positive selection carries an antagonistic character. Random mutations that may be a menace to intrinsic virus functionality are meshed during cycles of virus replication and propagation and are subsequently removed from the virus population leading to conservation of the sequences involved. Astrovirus protease, polymerase and VP34 are exemplary for this process of negative or rather purifying selection. Positive selection of substitutions on the other hand occurs in response to environmental changes and hence is also designated as molecular adaptation or adaptive molecular evolution. Obviously, sites prone to positive selection may be expected at domains in charge of communicative functionality like host range and immune response. It is therefore not surprising that sites belonging to the predicted serotypic epitope and to the putative RGD site for cell attachment in astrovirus ORF2 as well as to the two variable regions in astrovirus ORF1a display dN/dS values indicative for weak positive selection. The two clusters with dN/dS values >1 at the borders of VP25 may mark adaptive responses to maintain the structure of the virus spike protein allowing variability in the central part of VP25 that carries the serotypic epitope. The range of neutral evolution (0.3 < dN/dS < 1) is not very popular in astrovirus ORFs indicating that during evolution astrovirus has reached equilibrium between purifying selection and molecular adaptation.

Our attempts to correlate positively selected sites with virus functions also resulted in the identification of an RGD recognition motif for cellular attachment present in ORF2. In all astrovirus species (except Turkey1 isolate), an integrin recognition motif is found in ORF2. One of these (NGR) is located near the N-terminus. Bass and Qui [7] have shown that the aminoterminal 70 amino acids of the astrovirus ORF2 polyprotein can be deleted without consequences for virus assembly. Remarkably, they demonstrated this property for human astrovirus serotype 1 being the only species with all three integrin-binding motifs present in the sequence. Data on the process of cell entry by astrovirus have not been reported since 1992 [33]. Although experimental support is lacking, we tend to propose that integrin recognition plays a key role in astrovirus to target cell attachment.

## Methods

### Phylogenetic Analysis by Maximum Likelihood (PAML)

**Figure 4:**
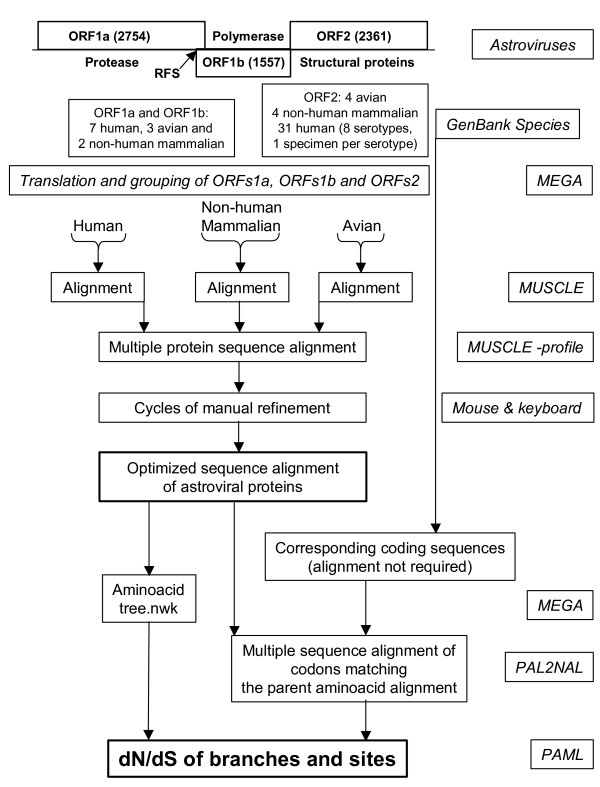
**Routing of astrovirus sequence data from GenBank to PAML input data files**. Main features of astrovirus genomes are given at the top. Numbers in parentheses indicate the length of an ORF in nucleotides. RFS points to the ribosomal frame shift signal between ORF1a and ORF1b. In GenBank, the number of complete ORF2 sequences surpasses those of ORF1a or ORF1b. At the ultimate right side, resources are boxed to indicate the actions undertaken and results obtained.

Sequences of astroviruses were obtained from GenBank and annotated previously [10]. Recently obtained sequences of mink [3] and human serotypes 4 and 5 [34] were also included in the present study. GenBank accession numbers are mentioned in Table 1 (Results section). The genomes of the astrovirus species include many human species with high sequence homology and few non-human mammalian and avian species, which are very divergent. Initial charges of PAML 3.14 for the estimation of (non-) synonymous rates of mutation in astrovirus genes demonstrated the need of a proper alignment of astrovirus sequences to avoid unconnected Markov chains, likelihood convergence problems and a collapse of the substitution model. Therefore, a novel strategy was developed to obtain properly aligned astrovirus sequences (Fig 4). In order to avoid overrepresentation of human astrovirus species (ORF2 analyses only), we included one specimen per human serotype except for human serotype 1. Besides a few point mutations, the human virus species Z25771 differs from L23513 (and from the other astrovirus species as well) by the presence of the oligopeptide that was reported to be deleted by cell culture adaptation of astrovirus [25]. Multiple sequence comparison by log-expectation (MUSCLE) [35] was used for the alignment of the polypeptide chains encoded by each astrovirus reading frame. A multi-step procedure was used. Firstly, three separate protein alignments were generated from the human, non-human mammalian and avian virus species, respectively. Secondly, these three alignments were combined into a single alignment file for each astrovirus ORF by means of the profile-profile operation of MUSCLE. Thirdly, refinements were introduced manually (BioEdit) [36] with special attention to sites near alignment gaps. An amino acid neighbor-joining tree was constructed in MEGA [37] and stored in newick format.

The PAL2NAL program was used for the conversion of a sequence alignment of proteins into the corresponding codon-based nucleotide alignment [38] that in turn was used for input into the CODEML module of PAML3.14 [11]. Most CODEML settings have been adapted from the small lysozyme data set of Yang [39]. The parameter-richness of the substitution model was effectively reduced by choosing F3x4 for CodonFreq and by attaching foreground labels to tip branches of the input tree instead of allowing a free determination of all these parameters. The choice F3x4 specifies the use of the correct nucleotide frequency for each of the different codon positions in the ORF involved [21]. Tree and branch labels were inspected for their integrity (TreeViewX) [40]. In order to apply the site models of CODEML, the parameter "model" was set to zero and "NSsites" was set to 0, 1, 2, 7 and 8 specifying different models of dN/dS variation to run in one batch [41].

This multi-step MUSCLE procedure for optimal sequence alignment required considerably less manual refinements than a one-step ClustalW alignment. It takes the PAL2NAL program only a few seconds to convert a collection of nucleotide sequences into a multiple sequence alignment of codons corresponding to the parent amino acid alignment. A single charge of 200 PAML iterations takes 4 to 24 hours of calculation on a P4/2.8 GHz with 504 MB of RAM under XP/SP1 depending on the length of the alignment and the number of species. Values of dN and dS in the branch models and of the ratio dN/dS in the site models of CODEML represent the number of (non-) synonymous substitutions per (non-) synonymous site corrected for multiple hits at the same site according to the PAML model in charge.

Server support and program downloads were received from CBS [42], PROSITE [43], PAML [44], MUSCLE [45] and PAL2NAL [46].

## Competing interests

The author(s) declare that they have no competing interests.

## Authors' contributions

FJvH carried out the computer analyses. All authors participated in writing the manuscript. VVL and BB are the principal investigators.
